# Sulphur Waters on the Secretory Organs

**Published:** 1844-03

**Authors:** 


*Sulphur Waters on the Secretory Organs.*—Between the action of mercury and the more powerful of the sulphur waters on the organic system, the most striking similarity exists. Dr. Armstrong long since remarked the resemblance between mercury and the sulphur waters of Europe, and confidently expressed the opinion that the latter are equally as powerful as the former, in their action upon the secretory organs; and with this very important difference, that while the long-continued use of mercury in chronic diseases, generally breaks up the strength, that of the sulphur waters generally renovates the whole system. Mercury has heretofore, by common consent, been regarded as the most powerful alterative we possess. I am not prepared to dispute this high claim of the medicine, but this much I will assert, as a matter of professional experience, that sulphur water, in my hands, has proved an *alterative* quite as certain in its effects as mercury, though somewhat slower in its operation. Not only so, I believe it to be far better adapted than mercury to a large circle of cases in which glandular obstructions and chronic inflammations are to be subdued. If the claims of the two remedies for preference, were otherwise near equal, the great advantage on the score of safety from the sulphur water, would give it immense preference over its rival. Numerous cases present themselves, however, in which they are used in conjunction to great advantage; where this becomes necessary, however, I have, as a general rule of practice, found it best not to continue the mercury longer than six or eight days: nor is it often necessary to use it continually during that period.—*Boston Med. and Surg. Jour.*, Feb., 1844.

